# One-Pot Method for Multifunctional Yolk Structured Nanocomposites with N-doped Carbon Shell Using Polydopamine as Precursor

**DOI:** 10.1186/s11671-016-1425-6

**Published:** 2016-04-19

**Authors:** Yanwei Zhang, Min Zhang, Lei Ding, Yongtao Wang, Jingli Xu

**Affiliations:** College of Chemistry and Chemical Engineering, Shanghai University of Engineering Science, Shanghai, 201620 China

**Keywords:** One-pot, N-doped, Yolk-shell

## Abstract

Herein, we reported a facile method to prepared uniform yolk like nanocomposites with well-defined N-doped carbon shell (C), in which the cores@SiO_2_@polydopamine (Pdop) were used as the sacrificed template. Typically, inherited from the functional Au core, the yolk particles presented excellent catalytic activities.

**Electronic supplementary material:**

The online version of this article (doi:10.1186/s11671-016-1425-6) contains supplementary material, which is available to authorized users.

## Background

Rattle-type or yolk-shell nanostructures have gained much attention, because they exhibited great potential applications in biomedicine [1], catalysis [2–4], nano-reactor [5, 6], and lithium-ion battery [7, 8], etc. Among them, the newly emerged Core@N-doped carbon yolk-shell structures have triggered great interest to scientists. Such a N-doped carbon shell functioned as a barrier to prevent the encapsulated nanoparticle from coalescence. Furthermore, the incorporated nitrogen atoms can be considered a tool for tuning the carbon properties, which enlarged the application of the carbon material, including electrodes for oxygen reduction reactions [9], metal-free catalysis [10], and CO_2_ capture [11, 12].

One-pot approach represents a green chemical strategy to simplify the synthesis process of core-shell-shell (CSS) nanostructures, and the template process was the most common and efficient method to fabricate these yolk-carbon shell structured nanomaterials from the CSS nanostructures. Thus, the technology combining the one-pot approach with the template process to obtain the yolk-like structured nanomaterials was highly desired by the scientist. Recently, the extension of the Stöber method for the synthesis of resorcinol-formaldehyde (RF) polymer nanospheres opens a novel pathway for synthetic strategies in the facile preparation of RF based core-shell and core@carbon yolk shell nanospheres. For example, Fuertes and coworkers reported a one-step Stöber method to synthesize RF@silica and carbon capsule structures [13]. Furthermore, Liu et al. reported one-step Stöber approach to produce uniform Au(Ag)-silica-polymer spheres with a core-shell-shell structure as templates for Au(Ag)@void@C yolk-shell nanostructures [14, 15]. Shao synthesized the magnetic rattle-type carbon nanospheres using the similar strategy [16]. These greatly widen the application of the RF Stöber method. However, a lack of nitrogen heteroatoms in RF results in an absence of electroactive nitrogen in the final carbon nanocomposites. Moreover, the strongly carcinogenic phenol/formaldehyde would do harm both to humans and the environment. Therefore, it is desirable to explore new polymer analogues that feature low toxicity, and the presence of heteroatom within a framework to prepare carbon nanocomposites for practical applications.

In contrast with phenol/formaldehyde, dopamine is nontoxic, widespread, and a sustainable resource. It contains carbon and nitrogen atoms and is well-known for its chelating capability with many types of metal ions. More importantly, the presence of nitrogen heteroatoms in carbon nanomaterials can strongly enhance the materials’ electrochemical performance [17–24]. Recently, Au@void@C was synthesized from Au@SiO_2_@Pdop, which exhibit high catalytic ability and stability in the reduction of 4-nitrophenol [25]. The iron oxide@void@C yolk-shell structure was also designed from the iron oxide@SiO_2_@Pdop for lithium batteries [26]. More recently, the MnO_2_@void@C yolk-shell nanorods with manganese oxide core and N-doped carbon shell have been constructed using a facile sol-gel method, which exhibit excellent performance in lithium batteries [27]. However, fabrication of core-silica-polydopamine (carbon precursor) involves multiple steps that are time consuming and energy wasting. Hence, a one-pot, effective, and general approach to synthesize CSS templates for yolk-carbon shell nanostructure production is still strongly needed.

Recently, Lu etc have reported that dopamine can be directly polymerized into monodisperse submicrometer spheres in a mixture containing water, ethanol, and ammonia at room temperature [28]. Inspired by this work, we have presented a one-pot strategy for the preparation yolk like nanocomposite with N-doped carbon shell by the extended Stöber method. By varying the core shape, the spherical, spindle, and wire-like structures were achieved. Typically, inherited from the functional Au core, the yolk particles presented excellent catalytic activities.

## Methods

### Materials

TEOS, HAuCl_4_, and Fe(acac)_3_ were purchased from Energy Chemical; Dopamine purchased from Alfa Aesar; ammonia solution (25–28 %), ethanol, sodium citrate, polyvinylpyrrolidone (PVP), ethylene glycol (EG), diethylene glycol (DEG), triethylene glycol, AgNO_3_, and NaCl were purchased from Sinopharm Chemical Reagent Co. (Shanghai, China). All the reagents were used without further purification. Deionized water was used throughout the experiments.

### Synthesis of Au Nanoparticles

Briefly, 30 mL of deionized water was magnetic stirred and 4.5 mL of 5 mg.mL^-1^ HAuCl_4_ solution was added. The mixed solution was stirred until boiling point was reached. Then, 1 mL of 3.3 wt% sodium citrate solution was added rapidly and the system was refluxed for 30 min. When the resultant colloid was cooled to room temperature, 10 mg PVP was added. The resultant was stirred for 24 h to allow complete adsorption of the polymer on the gold surface. After that, the solution was centrifuged (9500 rpm; 20 min) and the supernatant was removed. The volume of the concentrated colloid was then adjusted to 4 mL by dilution with deionized water.

### Synthesis of Au@SiO_2_@Pdop

Four milliliters of as-prepared gold nanoparticles in water was dispersed in 40 mL ethanol, then 1 mL ammonia aqueous solution (32 wt%) was added, after 5 min stirring, 0.2 mL TEOS was added. After that, the reaction mixture was stirred for 12 h at room temperature. Then, 200 mg dopamine was added and stirred for 24 h again. After that, the brown solid product was collected by centrifugation, washed with water and ethanol several times, and air-dried at 50 °C for 5 h.

### Synthesis of Au@void@C yolk-shell

Au@SiO_2_@Pdop was carbonized under N_2_ atmosphere at 500 °C for 5 h with a heating rate of 10 °C/min using tube furnace. Then, to remove the SiO_2_ in Au@SiO_2_@C, the as-synthesized powder was added into the mixture of deionized water (30 mL) and ammonia (10 mL) and then transferred into a Teflon-lined stainless steel autoclave. The autoclave was maintained at 140 °C for 12 h and cooled to room temperature. The Au@void@C yolk-shell particles was collected by centrifuging and washed with deionized water and ethanol several times and air-dried at 50 °C for 5 h. Then, Au@void@C was obtained.

### Synthesis of Cores@void@C yolk shell

The cores were Fe_3_O_4_, α-Fe_2_O_3_, Ag nanowires (Ag NWs), and CNTs/Fe_3_O_4_. Cores@void@C were obtained in a similar manner with the synthesis procedures of Au@void@C. The experimental details are shown in the supporting information.

### Catalytic properties of the Au@void@C composites

The reduction of methylene blue (MB) by NaBH_4_ was chosen as a model reaction for the testing catalytic efficiency of the Au@void@C nanocomposites. A given amount of the magnetic catalysts was added into a solution with MB (5 mL, 50 mg/L). After that, an aqueous solution of NaBH_4_ (1 mL, 0.4 mol/L) was rapidly injected at room temperature with stirring. The color of the mixture gradually vanished, indicating the reduction of the MB dye. Changes in the concentration of MB were monitored by examining the variations in the maximal UV-Vis absorption at 665 nm. After the catalytic reaction was completed, the nanocatalysts were separated by centrifugation and then repeated for the catalytic reaction. The recyclability of the nanoparticle catalysis was determined by measuring the maximal UV-Vis absorption of MB at the end of each catalytic degradation reaction.

### Characterization

The morphology of cores@void@C was observed using a scanning electron microscope (SEM, Hitachi S-8000, Japan) in a secondary electron scattering mode at 5 kV and a transmission electron microscope (TEM). X-Ray powder diffraction (XRD) patterns of the products were recorded with a Rigaku D/max-γB diffractometer equipped with a rotating anode and a Cu Kα source (*l* = 0.154 nm). The date of energy-dispersive X-ray spectrometer (EDS) data was obtained on a JEOL JEM 2010 electron microscope at an accelerating voltage of 200 kV. The surface area and mesoporous volume of hollow carbon spheres were measured by nitrogen physisorption measurements (micromeritics, ASAP 2460).

## Results and Discussion

**Scheme 1:**
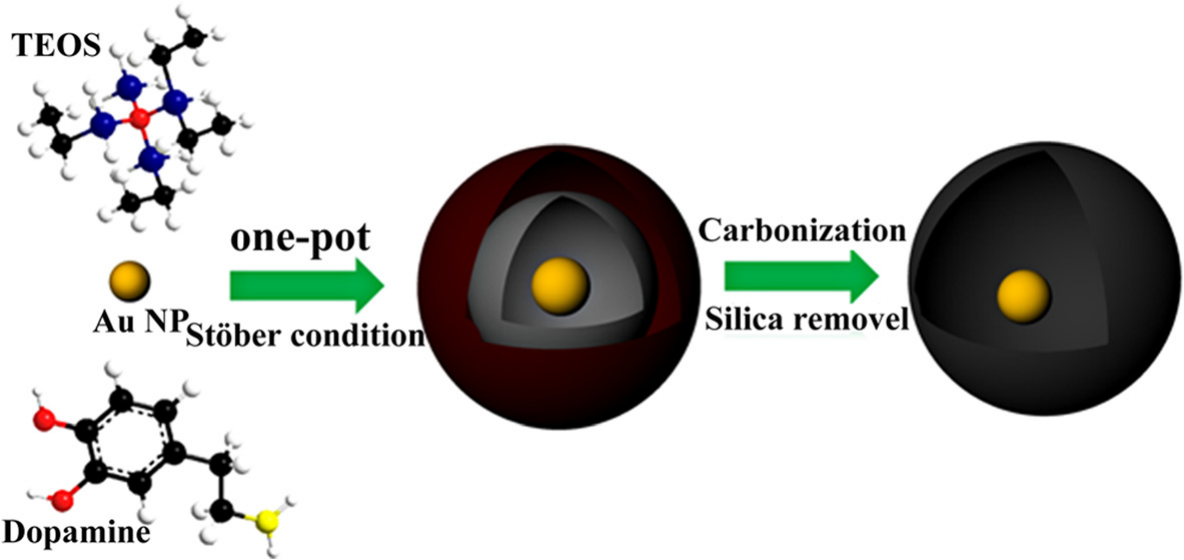
The synthesis of Au@SiO_2_@Pdop and Au@void@C

As shown in Scheme 1, we provide a facile route to form CSS nanostructures. Firstly, the Au@SiO_2_@Pdop can be easily obtained under Stöber reaction conditions with one-pot reaction. Then, the Au@void@C yolk-shell nanostructure was obtained after carbonization of the Pdop in N_2_ and followed by hydrothermal reaction with ammonium solution.

**Fig. 1:**
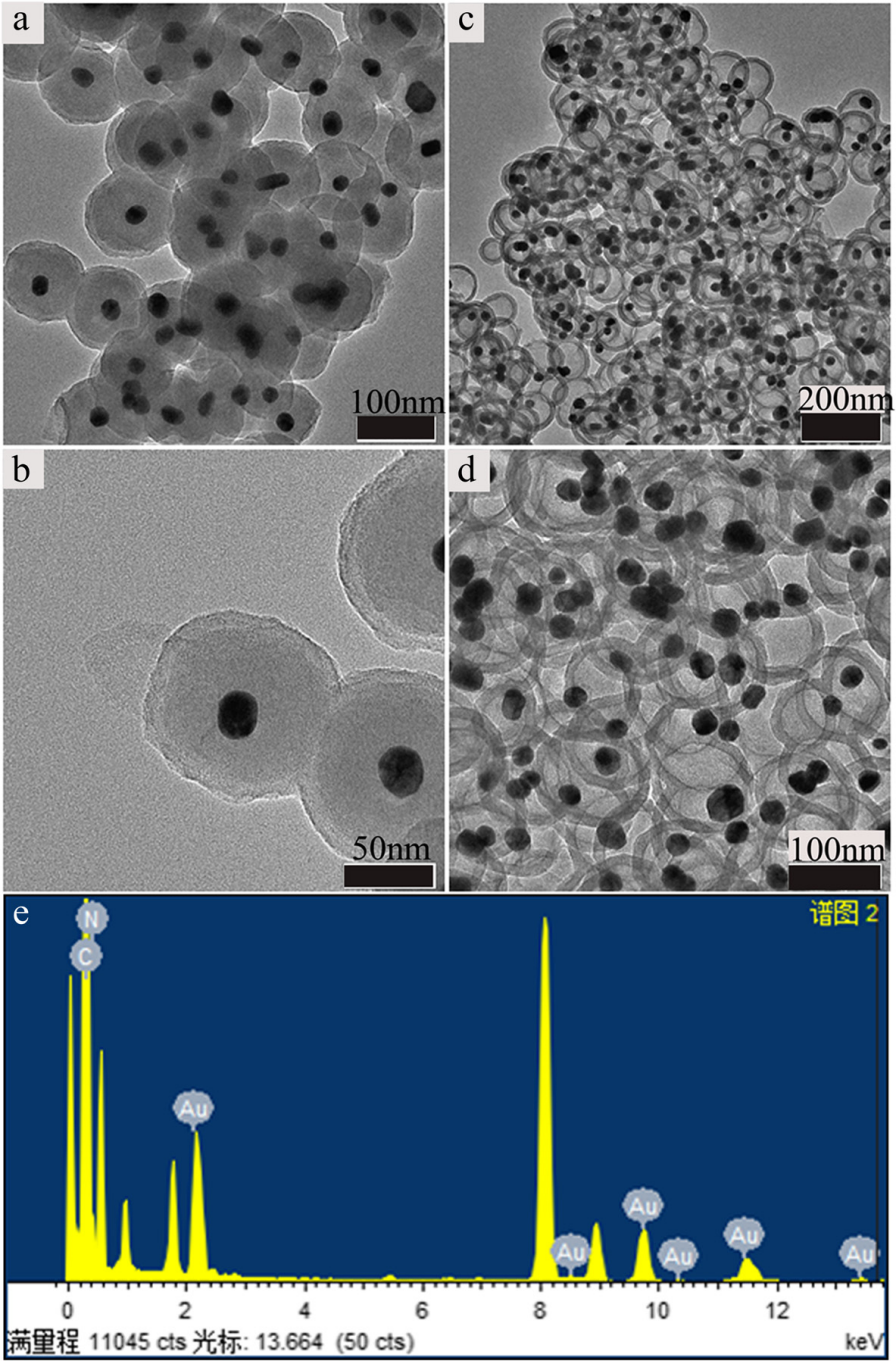
The TEM images of core–shell microporous carbon nanocomposites. Au@SiO_2_@Pdop (**a**, **b**) and Au@Void@C (**c**, **d**). Energy-disperse X-ray spectrum (EDS) of Au@void@C (**e**)

As for the synthesis of cores@SiO_2_@Pdop, the ratio of ethanol/water was the key parameter to form the CSS structures. It is beneficial for obtaining the CSS structures in the higher ratio of ethanol and water, which is due to that the middle SiO_2_ shell can be well coated on the core materials to avoid the formation of SiO_2_ spheres under this condition. Herein, the ratio of ethanol and water (10:1) was firstly selected to evaluate its feasibility. The test results indicate that the hollow carbon capsules without Au nanoparticles (Additional file 1: Figure S1(c,d)) were also observed, which is due to that the addition of TEOS is so quick that the silica sphere was formed in the TEOS Stöber process (Additional file 1: Figure S1(a,b)). This can be avoided by increasing the ratio of ethanol/water, or controlling the addition speed of TEOS. When we further increase the ratio of the ethanol and water to 15:1, the Au@SiO_2_@Pdop and Au@void@C were well obtained, which is shown on Fig. 1. From the TEM images, and the SEM images of broken particles (Additional file 1: Figure S2(e,f)) of an individual yolk-shell nanostructure, it is clear that the yolk-shell nanostructure of a carbon shell is encircling one metal nanoparticle. TEM image reveals that 113 nm Au@SiO_2_@Pdop particles and 120 nm Au@void@C particles with gold cores of ~20 nm are uniform and dispersed (Fig. 1a, b, Additional file 1: Figure S2). Here, the hollow void diameter is about 91 nm, which is consistent with the size of the silica particle in the core-shell-shell template. The carbon shell is about 15 nm thick. In addition, the energy-dispersive X-ray (EDX) spectrum of the nanostructures (Fig. 1e) clearly identifies the peaks of C, N, and Au. To further study the ratio of ethanol and water to influence on the formation of CSS structure, the ratio of ethanol and water (20:1) was also explored. The test result indicates that the polymerization speed of dopamine is so slow that no obvious phenomenon was observed after adding the dopamine for 24 h. Thus, combing the abovementioned, the ratio of ethanol and water (15:1) will be selected in synthesizing the cores@SiO_2_@Pdop in the following experiments.

**Fig. 2:**
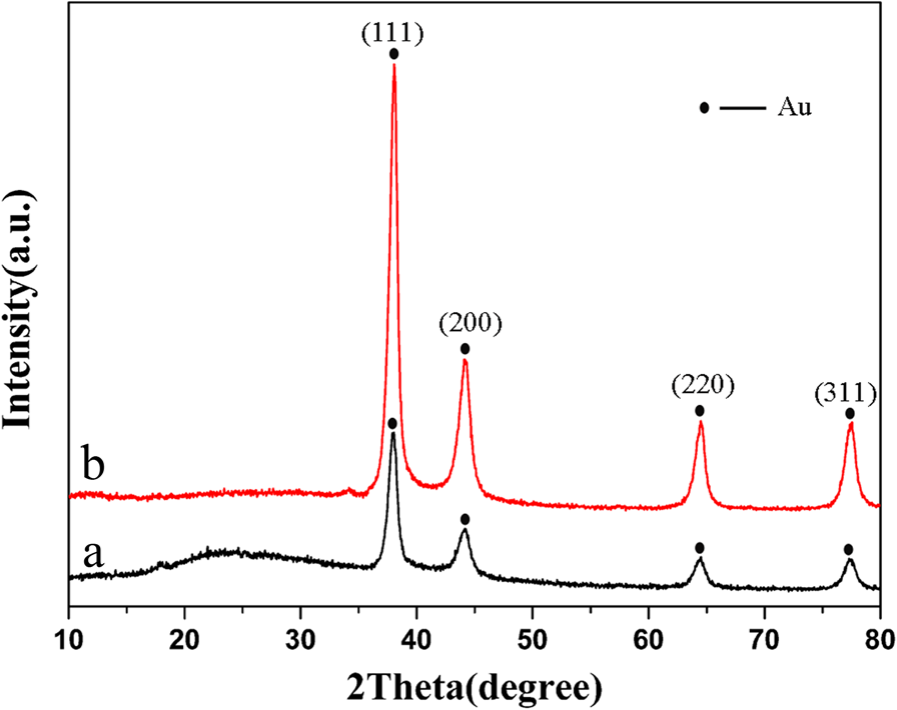
XRD patterns of Au@SiO_2_@Pdop (**a**) and Au@void@C (**b**)

An X-ray diffraction (XRD) pattern of Au@SiO_2_@Pdop (Fig. 2a) is indexed to Au, and the obvious broad peaks at 22° for the SiO_2_ is observed, indicating the SiO_2_ layer is amorphous. The XRD pattern (Fig. 2b) indicates that the Au core remained after calcination in nitrogen atmosphere. It is worth noting that the peak intensity of the amorphous peak cantered about 22°C was decreased which indicates that the silica layer was successfully removed.

**Fig. 3:**
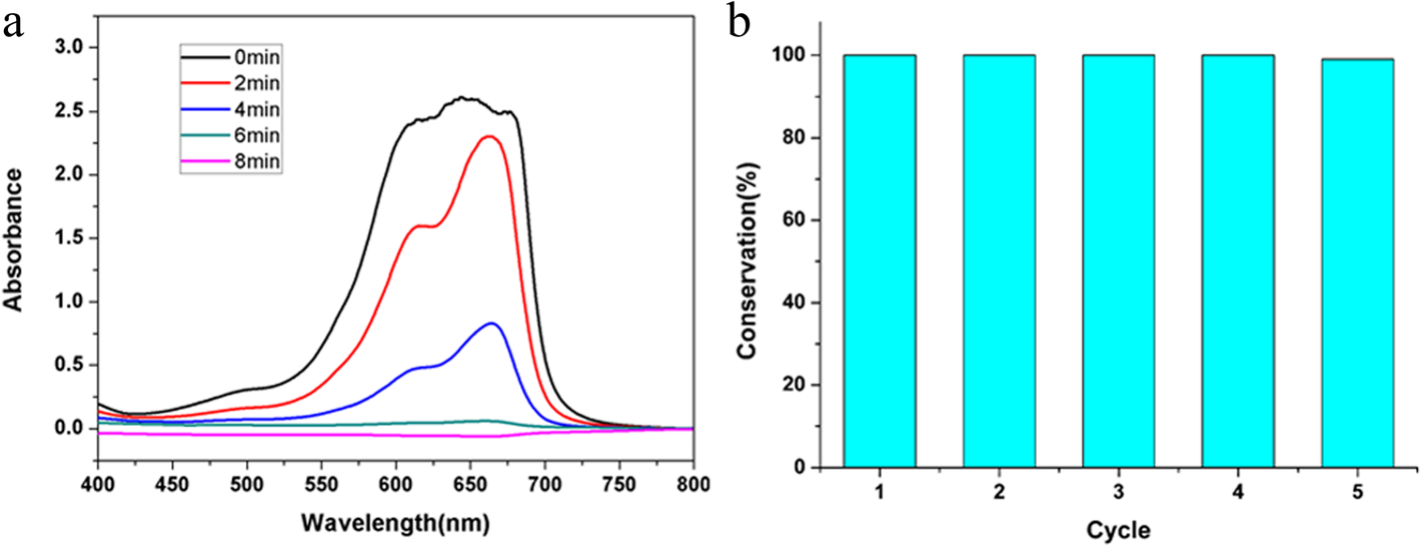
UV-Vis absorption spectra of MB during the reduction catalyzed by Au@void@C composite (**a**) and the recyclability of the Au@void@C as the catalyst for the reduction of MB with NaBH_4_ (**b**)

It has been experimentally demonstrated that Au NPs have high catalytic activity in reduction reactions of nitrophenols, hydrogenation, and CO oxidation [29–31]. To evaluate the catalytic activities of the yolk shell of Au@void@C nanocomposites, the reduction of MB by NaBH_4_ was selected as a model system. Without the Au catalyst, the reduction of MB proceeded at a slow speed with addition of NaBH_4_; the color of the MB solution endured great change, but does not completely disappear in 72 h. When a trace amount of Au@void@C was added into the mixture of NaBH_4_ and MB, the blue mixture became transparent within 8 min, which indicated that the MB was degraded completely (Fig. 3a). The apparent rate constant *k* calculated from the ln(C/C0) versus time plot (Additional file 1: Figure S3) was 0.018 s^−1^, which shows the excellent catalytic activity of Au@void@C on the MB reduction. Figure 3b shows the conversion for each run which was measured by UV/Vis spectroscopy. For Au@void@C nanocomposites, the reduction of MB drops slightly after each cycle, and it decreased gradually in subsequent runs to 99 % at run 5.

**Fig. 4:**
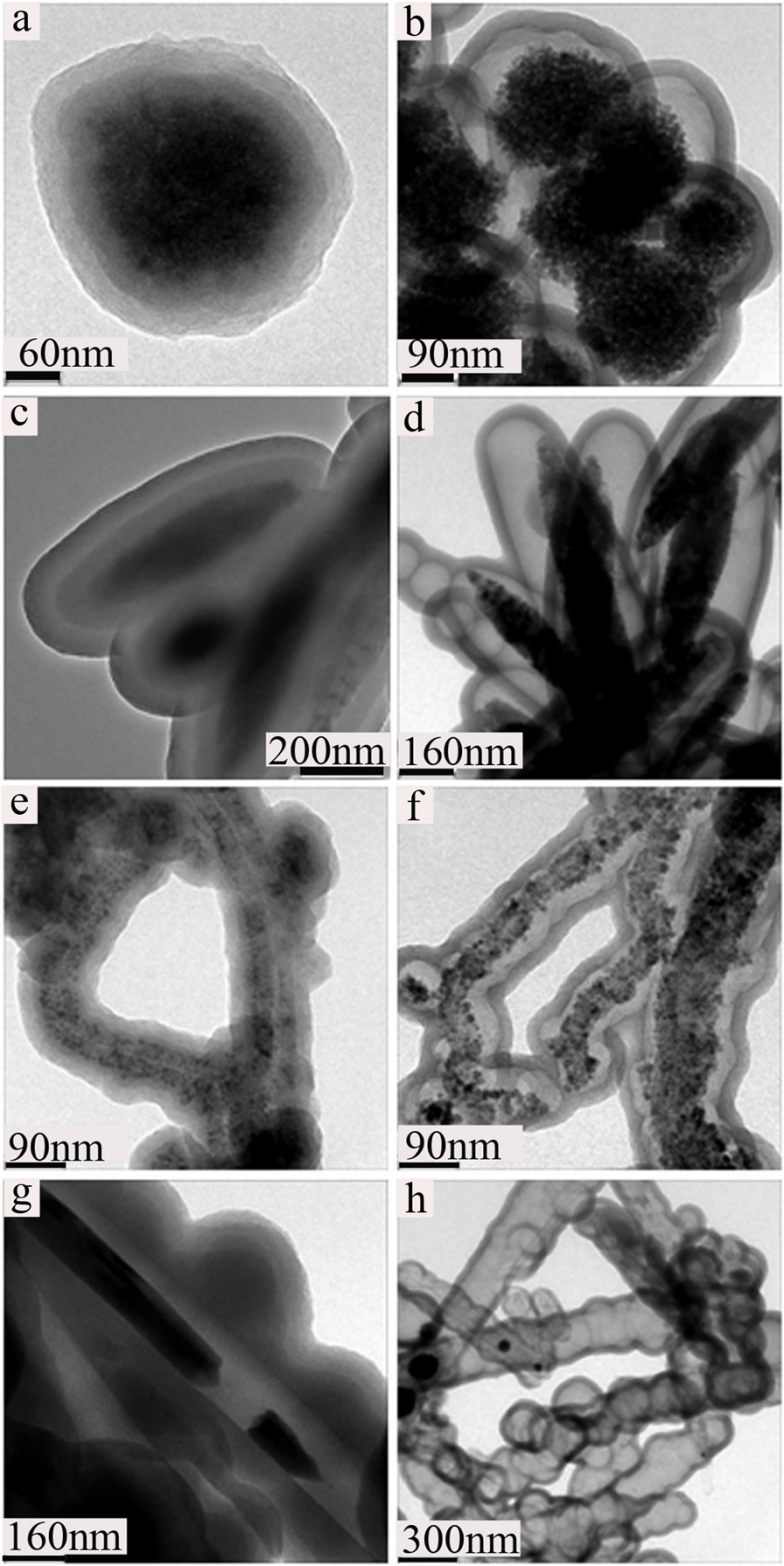
TEM images of core–shell and yolk shell functional nanocomposites. Fe_3_O_4_@SiO_2_@Pdop (**a**) and Fe_3_O_4_@void@C (**b**). α-Fe_2_O_3_@SiO_2_@Pdop (**c**) and α-Fe_2_O_3_@void@C (**d**). CNTs@Fe_3_O_4_@SiO_2_@Pdop (**e**) and CNTs@Fe_3_O_4_@void@C (**f**) Ag@SiO_2_@Pdop (**g**) and Ag@void@C (**h**)

The process developed here is a general and powerful method for coating colloidal particles of various compositions and shapes, including, for example, magnetic carbon nanotube [32, 33], superparamagnetic Fe_3_O_4_ spheres [34] and silver nanowires [35, 36] and rice-shaped α-Fe_2_O_3_ particles [37]. Using this synthetic method, we were able to coat Pdop@SiO_2_ and C@void on magnetic carbon nanotube, superparamagnetic Fe_3_O_4_ spheres and silver nanowires, α-Fe_2_O_3_ to synthesize multifunctional core-shell and yolk-shell composites, of which the SEM and TEM images were shown in Fig. 4, and Additional file 1: Figure S4, S5. Besides, it must be mentioned that in the coating process of the Ag@SiO_2_@Pdop (36 h), the core of Ag NWs was slowly etched by the ammonium solution, only a few Ag nanoparticles or nanorods exists in the multifunctional nanocable. Notably, the five core materials mentioned above are all hydrophilic, and can be easily deposited on the silica coating. All the results mentioned above clearly indicate that this novel approach can be widely applied to modify the hydrophilic surface of various nanomaterials. Additionally, it is not surprising to find that no silica coating could be realized on the functional core (hydrophobic surface) without surface modification due to incompatibility with the silica. As expected, mediation with proper surfactant of sufficient concentration (such as PVP) can also lead to a successful coating of silica on the functional core with hydrophobic surface; further work is underway. The as-prepared Pdop multifunctional composites could be converted into carbon composites after calcinations, which are believed to be able to find wide applications in, e.g., batteries, supercapacitors, and photocatalysts, etc.

## Conclusions

In summary, we have developed a versatile one-step methodology to produce yolk-shell structured nanocomposites using dopamine as the carbon source. The Au@void@C yolk-shell nanocomposites showed high catalytic ability and stability in the reduction of methylene blue. Moreover, the other kinds of yolk-shell carbon nanostructures such as magnetic carbon nanotube, Fe_3_O_4_, α-Fe_2_O_3_, and silver nanowires are obtained by carbonizing the Pdop and selectively etching the middle layer. The variety of advanced materials here presented has considerable interest due to their possible application in catalysis, drug delivery, electrochemistry, selective adsorption, and batteries, etc.

## Additional file

Additional file 1: Figure S1. The TEM images of core–shell Au@SiO_2_@Pdop (a, b) and Au@void@C(c, d) in the ratio of ethanol and water(10:1). **Figure S2.** The SEM imagines of Au(a,b), Au@SiO_2_@Pdop(c,d), Au@void@C(e,f). **Figure S3.** ln (C/C_0_) versus time for the reduction of MB. **Figure S4.** The SEM imagines of Fe_3_O_4_@SiO_2_@Pdop(a,b), Fe_3_O_4_@void@C(c,d), CNTs@Fe_3_O_4_@SiO_2_@Pdop(e,f), CNTs@Fe_3_O_4_@void@C(g,h), α-Fe_2_O_3_@SiO_2_@Pdop(I,j), α-Fe_2_O_3_@void@C(k,l), Ag@SiO_2_@Pdop(m,n), Ag@void@C(o,p) at different magnetification. **Figure S5** The TEM imagines of Fe_3_O_4_@SiO_2_@Pdop(a), Fe_3_O_4_@void@C(b), α-Fe_2_O_3_@SiO_2_@Pdop(c), α-Fe_2_O_3_@void@C(d), CNTs@Fe_3_O_4_@SiO_2_@Pdop(e), CNTs@Fe_3_O_4_@void@C(f), Ag@SiO_2_@Pdop(g), Ag@void@C(h) at higher magnetification. (DOCX 1310 kb)
