# Supplementary material for: One-Pot Method for Multifunctional Yolk Structured Nanocomposites with N-doped Carbon Shell Using Polydopamine as Precursor
Source: Nanoscale Res Lett. 2016 Apr 19;11:212. doi: 10.1186/s11671-016-1425-6 (PMC4837190; doi:10.1186/s11671-016-1425-6)
Supplement: Additional file 1: Figure S1. — The TEM images of core–shell Au@SiO2@Pdop (a, b) and Au@void@C(c, d) in the ratio of ethanol and water(10:1). Figure S2. The SEM imagines of Au(a,b), Au@SiO2@Pdop(c,d), Au@void@C(e,f). Figure S3. ln (C/C0) versus time for the reduction of MB. Figure S4. The SEM imagines of Fe3O4@SiO2@Pdop(a,b), Fe3O4@void@C(c,d), CNTs@Fe3O4@SiO2@Pdop(e,f), CNTs@Fe3O4@void@C(g,h), α-Fe2O3@SiO2@Pdop(I,j), α-Fe2O3@void@C(k,l), Ag@SiO2@Pdop(m,n), Ag@void@C(o,p) at different magnetification. Figure S5 The TEM imagines of Fe3O4@SiO2@Pdop(a), Fe3O4@void@C(b), α-Fe2O3@SiO2@Pdop(c), α-Fe2O3@void@C(d), CNTs@Fe3O4@SiO2@Pdop(e), CNTs@Fe3O4@void@C(f), Ag@SiO2@Pdop(g), Ag@void@C(h) at higher magnetification. (DOCX 1310 kb) [file 11671_2016_1425_MOESM1_ESM.docx]

**One-Pot Method for Multifunctional Yolk Structured Nanocomposites with N-doped Carbon Shell Using Polydopamine as Precursor**

Yanwei Zhang, Min Zhang,* Lei Ding, Yongtao Wang, Jingli Xu*

College of Chemistry and Chemical Engineering, Shanghai University of Engineering Science, Shanghai 201620, China. E-mail: [zhangmin@sues.edu.cn;](mailto:.zhangmin@sues.edu.cn;) [xujingli@sues.edu.cn](mailto:xujingli@sues.edu.cn)

**Synthesis of Fe_3_O_4_ NPs and Fe_3_O_4_@void@Carbon yolk-shell:**

The magnetic Fe_3_O_4_ were prepared according to the published literature. FeCl_3_.6H_2_O (1.08 g), NaOAc (4.0 g) were dissoved in EG (14 mL) and DEG (26 mL) under magnetic stirring. The obtained homogeneous yellow solution was transferred into a Teflon-lined stainless-steel autoclave. The autoclave was maintained at 200 °C for 15 h and cooled to room temperature. The Fe_3_O_4_ was collected by magnetic separation and washed with deionized water and ethanol several times and then dried under vacuum for 12 h. The Fe_3_O_4_@SiO_2_@pdop and Fe_3_O_4_@void@Cwere obtained in a similar manner with the synthesis procedures of Au@SiO_2_@pdop and Au@void@C.

**Synthesis of ellipsoid-shaped α-Fe_2_O_3_ and α-Fe_2_O_3_ @ void@Carbon yolk-shell:**

The α-Fe_2_O_3_ spindles were synthesized by aging a solution containing 680 mg of FeCl_3_.6H_2_O and 10 mg of NaH_2_PO_4_ dissolved in 80 mL of deionized water, then the solution was transferred into a Teflon-lined stainless-steel autoclave. The autoclave was maintained at 180 °C for 6 h. Then α-Fe_2_O_3_ spindles was collected by centrifuging and washed with deionized water and ethanol several times and air-dried at 50 °C for 5 h. The α-Fe_2_O_3_@SiO_2_@Pdop and α-Fe_2_O_3_@void@C were obtained in a similar manner with the synthesis procedures of Au@SiO_2_@Pdop and Au@void@C.

**Synthesis of Ag NWs and Ag NWs@ void@Carbon yolk-shell**

Ag NWs were synthesized according to the method reported previously. First, 2.93 g of PVP (K30) and 95 mL of glycerol were added into the flask. The composites were heated at 90 °C, when the PVP fully dissolved and the solution was cooled down to 50 °C. Then, 0.79 g AgNO_3_ powder was added. Subsequently, a solution containing 5 mL of glycerol, 29.5 mg of NaCl and 0.25 mL of H_2_O was added into the above solution and the solution temperature was raised from 50 °C to 210 °C in 25 min under gentle stirring (70 rpm). When the solution temperature reached 210 °C, the heating was stopped and the obtained gray-green solution was transfer immediately into a beaker. When the solution temperature returned to room temperature 100 mL deionized water was added. After stabilized for one week, upper layer solution was poured out to remove silver nanoparticles (Ag NPs) and a layer of sediment at the bottom of the beaker was collected and washed with ethanol three times to remove the rest PVP, and the Ag NWs were collected and dispersed into an aqueous solution of 10 mg/mL. Ag NWs@SiO_2_@pdop and Ag NWs@void@C were obtained in a similar manner with the synthesis procedures of Au@SiO_2_@pdop and Au@ void@C.

**Synthesis of CNTs/Fe_3_O_4_ and CNTs/Fe_3_O_4_@void@Carbon yolk-shell**

Briefly, 80mg CNTs, 120 mg of the iron precursor Fe(acac)_3_ and 30 mL triethylene glycol were added to the flask. The solution was sonicated for 30 min. Then, the mixed solution was heated to 278 °C under vigorous stirring and N_2_ protection and kept at reflux for 30 min. After cooling to room temperature, 30 mL ethanol was added to dilute the solution. Then the obtained composites were magnetically separated by a commercial magnet, which was washed with ethanol several times and air-dried at 50 °C for 5 h. CNTs/Fe_3_O_4_@SiO_2_@Pdop and CNTs/Fe_3_O_4_@void@C were obtained in a similar manner with the synthesis procedures of Au@SiO_2_@pdop and Au@ void@C.


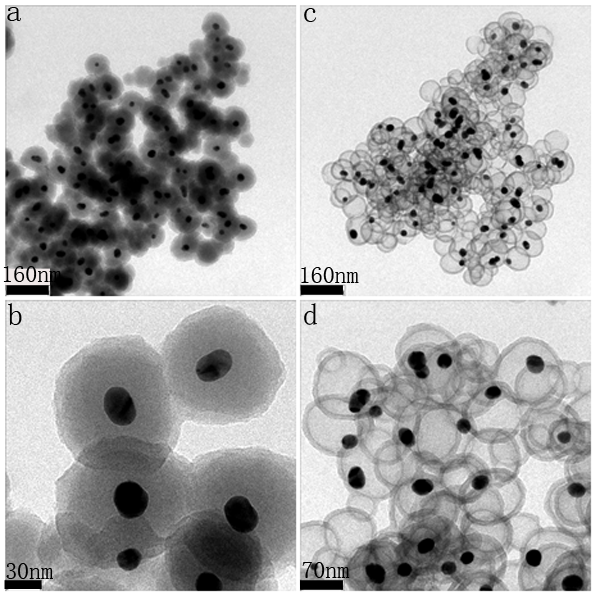


Figure S1 The TEM images of core–shell Au@SiO_2_@Pdop (a, b) and Au@void@C(c, d) in the ratio of ethanol and water(10:1)


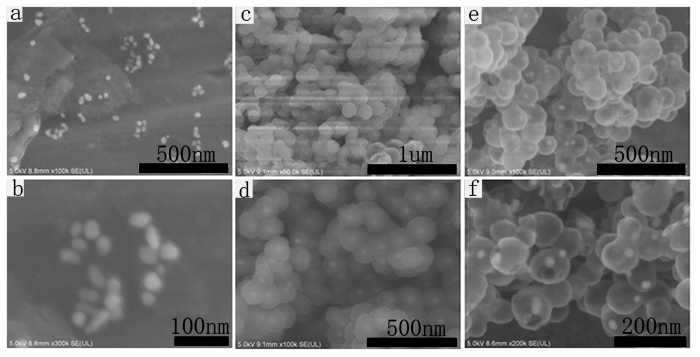


Figure S2 The SEM imagines of Au(a,b), Au@SiO_2_@Pdop(c,d), Au@void@C(e,f),


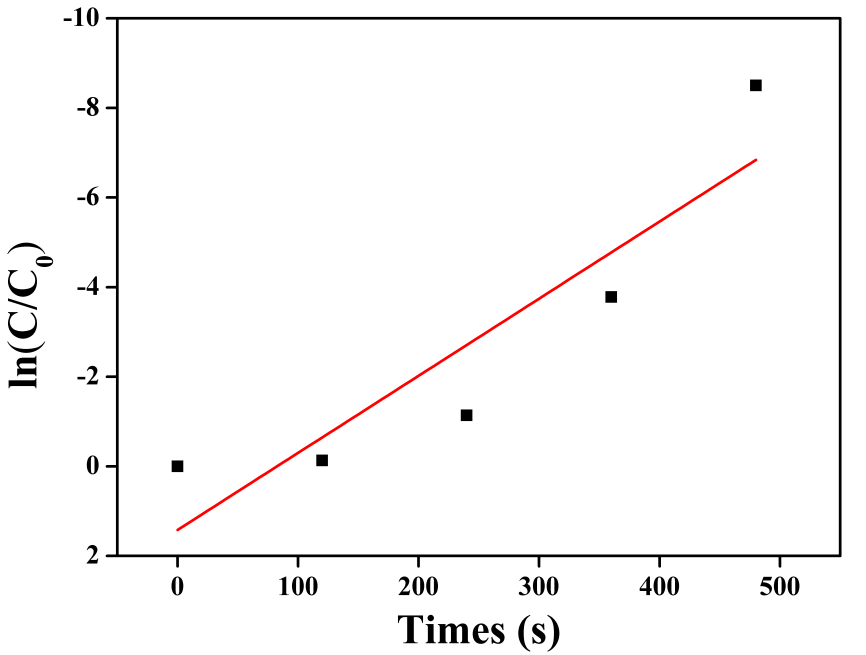


Figure S3 ln (C/C_0_) versus time for the reduction of MB


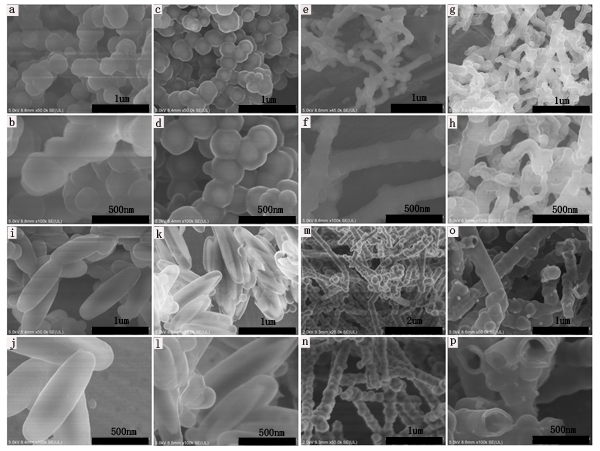


Figure S4 The SEM imagines of Fe_3_O_4_@SiO_2_@Pdop(a,b), Fe_3_O_4_@void@C(c,d), CNTs@Fe_3_O_4_@SiO_2_@Pdop(e,f), CNTs@Fe_3_O_4_@void@C(g,h), α-Fe_2_O_3_@SiO_2_@Pdop(I,j), α-Fe_2_O_3_@void@C(k,l), Ag@SiO_2_@Pdop(m,n), Ag@void@C(o,p) at different magnetification


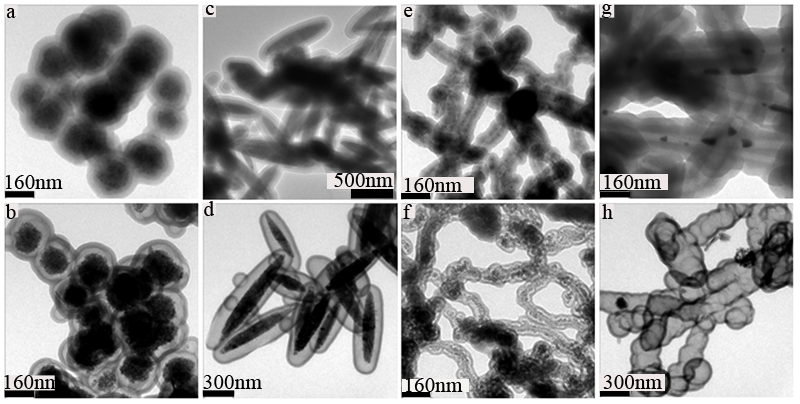


Figure S5 The TEM imagines of Fe_3_O_4_@SiO_2_@Pdop(a), Fe_3_O_4_@void@C(b), α-Fe_2_O_3_@SiO_2_@Pdop(c), α-Fe_2_O_3_@void@C(d), CNTs@Fe_3_O_4_@SiO_2_@Pdop(e), CNTs@Fe_3_O_4_@void@C(f), Ag@SiO_2_@Pdop(g), Ag@void@C(h) at higher magnetification
